# Impacts of Typhoon Soudelor (2015) on the water quality of Taipei, Taiwan

**DOI:** 10.1038/srep25228

**Published:** 2016-04-29

**Authors:** Hoda Fakour, Shang-Lien Lo, Tsair-Fuh Lin

**Affiliations:** 1 Graduate Institute of Environmental Engineering National Taiwan University No. 1, Sec. 4, Roosevelt Rd., Taipei 10617, Taiwan (R.O.C.); 2 Department of Environmental Engineering and Global Water Quality Research Center, National Cheng Kung University, Tainan City, Taiwan

## Abstract

Typhoon Soudelor was one of the strongest storms in the world in 2015. The category 5 hurricane made landfall in Taiwan on August 8, causing extensive damage and severe impacts on the environment. This paper describes the changes of trihalomethane (THM) concentrations in tap and drinking fountain water in selected typhoon-affected areas in Taipei before and after the typhoon. Samples were taken from water transmission mains at various distances from the local water treatment plant. The results showed that organic matter increased between pre- and post-typhoon periods with a greater proportion of aromatic compounds. Although drinking fountains showed moderately less total trihalomethane (TTHM) levels than that of tap water, the intake of high turbidity water considerably diminished the efficiency of their purification systems after the typhoon. The percentage distribution of THM species increased throughout the distribution network, probably due to a longer contact time between chlorine and the organic matter in the pipelines. After 2 to 5 min of boiling, THM reduction was considerable in all cases with the greater extent in post-typhoon samples. It is evident that extreme weather conditions may have a severe impact on water quality, and thus more cautious strategies should be adopted in such cases.

Typhoons, also called hurricanes or tropical cyclones, are one of the most catastrophic natural hazards, causing severe devastation in coastal regions. In addition to the high winds that directly disrupt infrastructures and housing^1^, typhoons can impact many ecosystem functions. Extensive rainfall can also induce the flow of flood waters into an estuary, leading to lower salinity and increased organic matter and nutrients in the estuary^2^.

Taiwan, a small sub-tropical island (36,000 km^2^), is one of the most typhoon-impacted regions in the world^3^. Typhoon Soudelor, the most intense tropical cyclone that developed in the Northern Hemisphere in 2015, formed in the middle of the Pacific Ocean on July 20, 2015, and became more vigorous when it was well east of Taiwan over the open water^4^. It was the world’s strongest storm in 2015, with maximum sustained winds of 180 mph, which is equivalent to a category 5 hurricane on the Saffir-Simpson hurricane wind scale^5^. More than 4 million customers lost power, according to < taipower.com>, which was the most severe typhoon-caused power outage record thus far in Taiwan.

High turbidity in Taipei city’s raw water and the poor quality of tap water in certain areas sparked waves of panic in the city during the typhoon event. Many people noticed yellow and brownish tap water that smelled like soil after the typhoon swept through Taiwan. The turbidity reading in the Xindian River, one of the main sources for drinking water in Taipei City, reached as high as 39,000 nephelometric turbidity unit (NTU)^6^.

High turbidity may affect public health. In a study conducted in 1997, Harvard University researchers found that hospital emergency room admissions rose in parallel to the turbidity level in drinking water of studied areas^7^. In addition, the largest outbreak of waterborne disease in the US, Milwaukee’s Cryptosporidium outbreak in 1993, was linked to high turbidity and high bacterial counts in source water that were caused by severe spring storms^8^.

Due to climate change, typhoons and extreme weather events are occurring more frequently^9^, resulting in high turbidity source water, usually in the thousands of NTUs, during the heavy rainy seasons. On August 23, 2000, Taiwan was hit by tropical storm Bilis, which caused serious flooding problems. The raw water turbidity at PingTsan Waterworks of the Taiwan Water Corporation was measured at up to 1200 NTU for over a week and at several hundred NTUs for over a month. The total organic carbon (TOC) of the raw water also reached to more than 10 mg/L^10^.

High levels of organic matter in raw water negatively affect the coagulant and chlorine demand, which could cause an increase in the possibility of formation of disinfection by-products (DBPs), such as trihalomethanes (THMs), haloacetic acids (HAAs), and haloacetonitriles that might be harmful to human health^11^.

Several hundreds of DBPs exist in treated drinking water. THMs and HAAs are still of most concern, as they are good indicators of the overall DBPs in chlorinated water. Other DBPs, such as haloacetonitriles (HANs), chloropicrin and chlorinated furanones, are usually present at lower concentrations^12^.

THMs are the major by-products of chlorination with some evidence of a potential health hazard from long term exposure that often occurs from drinking water as a result of the chlorine treatment for disinfection purposes^13^. Chlorination is the most common disinfection process used in many countries, including Taiwan. In Taiwan, chloroform, a major THM species, has been found in disinfected water supplies^14^. Chlorinated DBPs are more common than other types, with THMs usually more dominant than HAAs in drinking water systems^15^.

In 2001, the USEPA set the maximum contaminant level (MCL) for THMs in drinking water at 80 μg/L with the declared intention of eventually lowering it to 40 μg/L, which is currently mandatory for systems applying a reduced monitoring^16^. Some countries have established even stricter standards for THMs. Germany has stablished a limit of 50 μg/L if the total THM (TTHM) level is less than 10 μg/L at the exit of waterworks. In the Netherlands the 90^th^ percentile value set at 25 μg/L with a maximum concentration of 50 μg/L^17^.

In addition, THMs may remain persistent in water distribution systems. The half-life of THM species ranges from 1 to 65 days, with chloroform having the highest permanence^18^,^19^. Moreover, in drinking water treatment processes, coagulation generally removes more HAA precursors than THMs because conventional coagulation treatment is not always able to satisfactorily reduce the THM precursor levels^20^.

Nguyen *et al.*^21^ identified significant inputs of terrestrially derived dissolved organic matter (DOM) during two storm events in South Korea. Hong *et al.*^22^ suggest that heavy rainfall induces proportionally more DBP precursor compounds than low-flow conditions, and thus significantly affects water quality characteristics.

An individual’s intake of water DPBs is usually estimated based on DBP concentrations in tap water or information from water treatment processes at purification plants, although this is insufficient to accurately quantify DBP exposure from drinking water. In the current study, we determined the effects of different approaches in processing of water for consumption, commonly used in households or workplaces, on drinking water DBP concentrations. Among different drinking water appliances, public water fountains have become very popular over the last decades, and increasingly more and more people have discovered the benefits that a fountain can provide. A drinking fountain, also called a water fountain, is a device which streams water into a basin or jets it into the air to supply drinking water. These public devices are most often seen in schools, playgrounds and parks. Modern indoor drinking fountains usually incorporate filters to remove impurities from the water. Even with their steady growth and popularity, drinking fountains are not without controversy. Many people choose public drinking fountains because they believe they are safer than tap water, but there is not much solid evidence to support this. Moreover, there is very little published literature on the effects of these newer devices on drinking water DBP levels, particularly under extreme weather conditions.

To shed light on the role of super typhoons on drinking water quality, this paper describes THM concentrations of tap and drinking fountain water throughout the water transmission network in selected typhoon-affected areas in Taipei City, Taiwan before and after typhoon Soudelor.

Because drinking water DBPs can also be associated with different tap water handling strategies, the effect of boiling on DBP concentrations was investigated as well. Despite numerous studies on the impacts of boiling on THM concentrations, there is no published literature regarding the effects of boiling in extreme weather conditions with high turbidity and organic matter loading in raw water intake. During the recent typhoon Soudelor event, many people complained to water companies about being unable to drink or take a bath because of the high murkiness of the water. Others purchased over-sized bottled water supplies and emptied supermarket shelves. For a few days, local authorities recommended boiling tap water due to public health concerns. The current study can generate very valuable information for use by water treatment authorities in devising effective protocols for reducing THM by-products under extreme weather conditions.

## Results

### Typhoon Soudelor Characteristics

Soudelor formed as a tropical depression near Pohnpei on July 29, 2015. The system strengthened slowly before starting rapid intensification on August 2. Soudelor made landfall on Saipan, causing extensive damage. Due to favourable environmental conditions, the typhoon further strengthened and reached its peak intensity of 215 km/h (130 mph) and an atmospheric pressure of 900 hPa (mbar; 26.58 in Hg) on August 3. The storm made its landfall in eastern Taiwan at 04:40 a.m. local time on August 8. By mid-morning, maximum sustained winds of 173 km/h (107 mph) were recorded by Taiwan’s Central Weather Bureau (CWB). The typhoon then moved inland through Eastern China and degraded to a tropical depression by August 9.

A study by Bae^23^ compared a river’s water quality during three different rainfall events in South Korea and showed that the changes of water quality due to rainfall events are dependent on the amount of precipitation and the temporal patterns of rainfall events.

**Figure 1:**
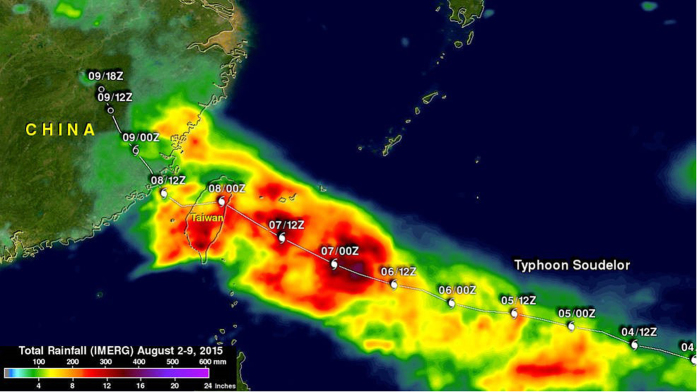
NASA generated rainfall map for typhoon Soudelor 2015 (Image Credits: SSAI/NASA/JAXA, Hal Pierce).

Figure 1 shows the NASA-generated rainfall map for the Taiwan and China area. This analysis covers the period from August 2–9, 2015, with rainfall from typhoon Soudelor within the area of this analysis after August 3, 2015.

Taipei reported 322.5 millimetres (12.70 inches) of rain at the city centre, while some other districts of the city had more than double that total. The Mao Kong observation site in the city’s Wenshan District reported 659.0 millimetres (25.94 inches) of rainfall^24^.

Taiping Mountain in eastern Taiwan’s Yilan County experienced the heaviest rain from the typhoon with peak accumulation at 1,334 mm (52.52 in). Rainfall in the Wulai District also reached 722 mm (28.4 in) in 24 hours, the second-highest on record after typhoon Matsa in 2005^25^.

Torrential rain and vigorous winds resulted in widespread damage and disruptions. The strongest winds with a peak intensity of 211 km/h (131 mph) were observed in mountainous areas in Northern Taiwan^26^. Subsequently, a contaminated Nanshi River, which is the primary water source of the Taipei-Keelung metropolitan area, overflowed. The river’s turbidity level increased very quickly, and the excess water overwhelmed the local water purification system. As a result, the water supply for approximately five million people in Taipei was polluted for the next several days.

### Water quality parameters

**Figure 2:**
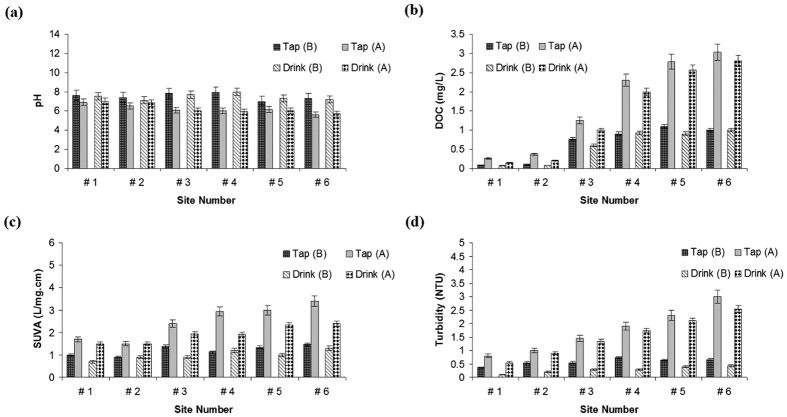
pH (**a**), DOC (**b**), SUVA (**c**), and turbidity (**d**) variation before (**B**) and after (**A**) typhoon Soudelor (2015) at the six studied sampling sites.

Figure 2 summarizes the average water quality parameters (dissolved organic matter (DOC), turbidity, pH, and specific ultraviolet absorbance (SUVA)) and their variations during pre- and post-typhoon phases in tap and drinking fountain water samples. While the pH levels decreased in post-typhoon samples, turbidity levels considerably increased after the typhoon event. The highest turbidity that the purification plant can handle is approximately 6,000 NTU, although water murkiness levels for the Xindian River reached as high as 39,000 NTU during the typhoon event, as reported by local authorities. Although drinking fountain water samples generally illustrated lower turbidity than tap waters, in some cases, the degree of turbidity overwhelmed the filtration system treatment efficiency (above the local standard of 2 NTU), resulting in the placement of warning labels on drinking water fountains.

The average DOC concentration in tap and drinking water samples in the pre-typhoon phase were 0.66 and 0.43 mg/L, respectively. DOC concentrations increased in post- typhoon samples to an average level of 1.72 and 1.39 mg/L for tap and drinking water, respectively.

The SUVA-inferred quality of the organic matter following the typhoon also changed, with a greater proportion of aromatic and higher molecular weight compounds following the storm event. Weishaar *et al.*^27^ suggested that SUVA_254_ is strongly correlated with some aromatic organic matter and could be a useful parameter for aromatic DOC.

### THMs concentrations for pre- and post-typhoon Soudelor (2015) conditions

Table 1 shows the mean concentration of TTHM (μg/L) in tap and drinking water samples collected before and after typhoon Soudelor (2015). The highest TTHM level for tap water in the pre-typhoon phase was 32.8 μg/L, which is far less than the MCL for drinking water. Similarly to SUVA and DOC distribution patterns (Fig. 2), the THM levels in tap water samples after the typhoon increased to a maximum level of 64.9 μg/L. The observation of higher THM levels in post-typhoon drinking fountain samples are in agreement with a study by Nguyen *et al.*^21^, in which higher DBP formation potentials (DBPFPs) were found for the storm and post-storm samples than for the baseline condition. However, our observation is different to that reported in Delpla *et al.*^28^, who showed that during heavy rainfall in northwestern Europe, the change in potable water DOC quality and quantity did not significantly alter the THM formation potential. The difference between the current study and Delpla *et al.*^28^ might be due to different sampling times and target area. In their study, sampling was conducted during the spring season from mid-water depth of rivers or streams. The quality and availability of organic matter are seasonally different. Thus, the impact of DOC on transport processes and its interaction with other compounds may vary throughout the year due to changes in temperature and DOC composition.

The concentrations of all forms of THMs gradually increased in accordance with the distance from the source water to the sampling point (Table 1) which might be due to the longer contact time between chlorine and organic matter in the pipeline and/or higher DOC levels, a major THM precursor, in the distribution system^29^.

### DBPs species distribution

**Figure 3:**
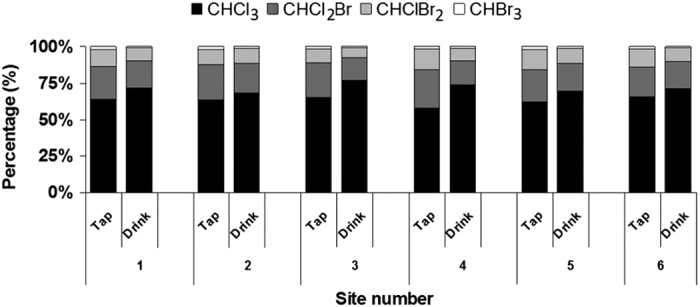
THM species distribution in tap and drinking water samples before the storm.

Although higher brominated THM species were observed in some of the drinking water samples from the post-typhoon phase (data not shown), the THM species distribution pattern was not significantly different before and after the typhoon event (P-value = 0.14). Figure 3 presents the general distribution pattern of THM species in pre-storm samples, where CHCl_3_ was the major THM species accounting for an average of 63.6% and 72.2% of the total THMs in tap and drinking water, respectively.

### Correlations of THMs with other parameters

Table 2 lists the correlation matrix for studied tap water quality parameters, with the THM concentrations, for a total of 48 observations with triplicate analyses in pre- and post-typhoon samples. While, in the pre-typhoon stage, only DOC and SUVA showed significant correlation with the THM level, there was a high correlation between DOC, SUVA, pH and turbidity in the post-typhoon phase (P < 0.05) indicating the presence of a higher molecular weight natural organic matter (NOM) fraction in intake water after the typhoon. Both Pearson’s and Spearman’s rank correlations follow the same trend, suggesting that the outliers are not substantially influencing the results. The findings are in agreement with the study by Kitis *et al.*^30^, in which strong correlations were observed between DBP formations and SUVA values of many NOM fractions obtained by various methods of physicochemical separation. It is known that a higher organic content of water requires higher chlorine doses and thus influences residual chlorine in the pipelines and consequent increases in the THM levels. In summary, for all studied samples, the THM indicators effectively increased after heavy storm conditions.

### Boiling strategy

**Figure 4:**
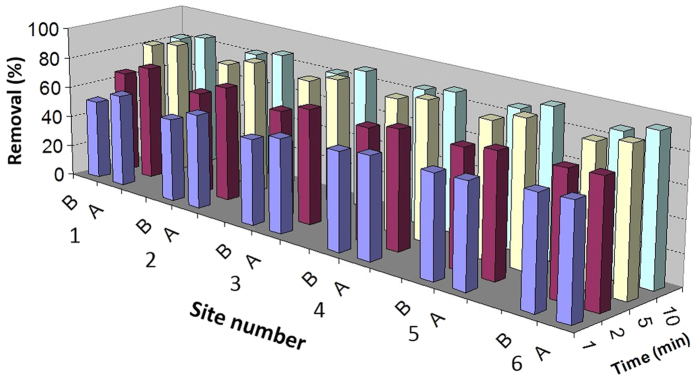
The effect of boiling time on the removal efficiency of TTHM in tap water samples before (**B**) and after (**A**) typhoon Soudelor (2015).

**Figure 5:**
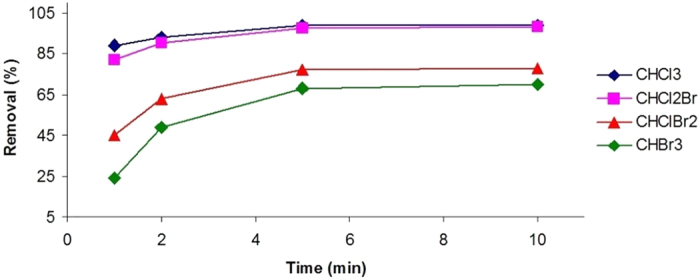
Effect of boiling time on removal (%) of each THM species in post-typhoon tap water samples.

Boiling significantly reduced the THM levels in post-typhoon tap water samples (Fig. 4). TTHM removals ranged from 51.5 to 89% and 60 to 96% for different boiling times in pre- and post-typhoon samples, respectively. Fifty percent or more of each species was removed following 2 min of boiling, whereas 70% or more of each THM was removed upon boiling post-typhoon samples for 5 min (Fig. 5). Chloroform showed the highest average removal percentage, even in less boiling time (i.e., 1 min).

The results are in agreement with those of Levesque *et al.*^31^, in which filtering and boiling tap water was shown to reduce the significant concentrations of THMs, and Wu *et al.*^32^, who observed an 83% reduction of chloroform after 5 minutes of boiling.

## Discussion

The impact of extreme weather conditions such as typhoons and heavy rainfall, on drinking water quality is considered an important issue of emerging concern, as the magnitude and frequency of such events are increasing in response to climate change^9^. In recent years, strong typhoons and hurricanes that have frequently hit Southeast Asia and the eastern United States are attributed to climate change. Low-lying areas covered with muddy waters contain a large quantity of clay and silt from soil erosion after heavy rains. During storm seasons, the turbidity of large rivers significantly increases. In early March of 1995, a heavy spring precipitation caused the turbidity to increase from 100 to over 1,000 NTUs in the Missouri River. In Taiwan, the number of typhoons during the 1990s was three times of that in the 1960s, and the turbidity in many rivers and reservoirs often exceeded 10,000 NTUs during storm seasons^33^.

Many relevant studies have been conducted to investigate the long-term changes of DBP precursors in drinking water sources. In contrast, changes in DBP precursors over short periods of time have been overlooked despite their significant impact on DOM characteristics in downstream reservoirs having long residence times. The rapid input of new terrestrial DOM sources from overflowed rivers may change the composition of DOM considerably in reservoirs used as drinking water sources. Using samples collected before and after three 1999 hurricanes, Paerl *et al.*^34^ showed that the DOC concentration in the Neuse River estuary, one of the largest tributaries of Pamlico Sound, increased about two times compared with pre-hurricane values. Despite recent progress, there is still a lack of understanding about the changes in DBP precursors and/or DOM characteristics during extreme weather conditions. In addition, it is important to determine the relationships between DOM quality and DBPFPs during storm conditions to conduct a real-time monitoring of drinking water sources.

In the current study, the impact of typhoon Soudelor (2015), on the drinking water quality and the THM concentrations in a selected typhoon-affected area in Taipei, Taiwan was studied using water samples collected before and after the typhoon event. Both tap and drinking water samples were considered in the sampling programme. The effect of boiling, as a common household water handling strategy, on the THM concentrations was also investigated.

Water quality parameters were considerably influenced by typhoon Soudelor. Lower pH was observed in post-typhoon samples of water distribution system. Having reacted with atmospheric carbon dioxide, rain is naturally acidic with an average pH level of approximately 5.6^35^. Increased surface levels reduced the transit time between rain falling onto the ground and the runoff entering surface water, resulting in less time for the pH modification associated with organic and inorganic effects. Hence, with increased surface water levels and runoff volumes, pH levels decline^36^. However, pH in heavy rain is not simply set by the transit times, as organic acid fluxes into surface waters may also decrease the pH, creating a more stressful environment as low pH values are often found in DOM-laden water. It has been shown that a considerable portion (often >50%) of DOM comprises of organic acids derived from humic compounds^37^.

Super typhoon Soudelor also led to a considerable rise in turbidity and organic matter concentrations in the drinking water from the distribution system.

During high turbidity events, the concentration of suspended particles increased in the source and treated water, and a similar increase is possibly observed throughout the distribution system compared with baseline conditions. Water passing through the distribution system may also show increased turbidity due to the re-suspension of fine particles settled over a long period of time, the breaking of pipe materials, or biofilms lining the walls of the pipes^38^.

Under storm conditions, soil particles and debris from industrial and residential areas can be washed into streams. Because of the large paved areas in urban regions, natural settling spots have vanished, and sediment is quickly transferred by storm drains to creeks and rivers. Therefore, water turbidity increases during runoff events as a result of erosion and overland flow.

High intake NOM levels may also influence the accumulation of organic matter onto corrosion sediments along the distribution system and adversely affect water quality. On the other hand, higher levels of organic matter in the source water after flood events necessitate more chlorine, and the level of THMs increases accordingly^39^.

An association between DOM quantity and quality with DBPFP has been previously reported in other studies conducted using samples collected under non-storm conditions^40^. Our results confirmed the fact that although there are different external DOM sources mixed with the inherent organic matter in raw water, DOC level and reactivity is still beneficial for estimating DBP levels, even during extreme weather conditions with relatively short time periods. DOC quality changed consistently at all sampling sites after the typhoon event, with a greater proportion of aromatic and higher molecular weight compounds. SUVA results indicate that raw water intake was composed mainly of humic substances after the typhoon, probably due to mixing with soil organic matter through landslides upstream. Delpla *et al.*^31^ showed a considerable rise (up to 158%) in organic matter concentration in potable water after a heavy rainfall in northwestern European. They also reported that DOC quality, in terms of SUVA, changed consistently during rainfall events, with a greater proportion of aromatic and higher molecular weight compounds following the beginning of rainfall.

While the current results are in agreement with some previous studies^33^,^41^, in which strong correlations were observed between DBP formations and SUVA values of many NOM fractions, they contradict with some others^42^,^43^, wherein SUVA did not correlate well with the formation of TTHM. This is likely because SUVA correlation with DBP formation is water specific, being stronger in waters with high SUVA values containing higher molecular weight NOM fractions^42^. This can be verified by the correlation matrix for water quality parameters and THM concentrations (Table 2), in which a stronger correlation between SUVA_254_ and THM was observed in samples after the typhoon compared with pre-storm samples, and SUVA values were higher after the storm (Fig. 2).

The change in water quality parameters significantly altered the THM levels for both tap and drinking water samples. Tap water THM levels are generally higher than drinking fountain water THMs, which points to the importance of using drinking fountains in households or workplaces, particularly in severe weather conditions.

Although the TTHM levels were generally lower in drinking fountain water than tap water, the typhoon-induced contamination of the intake water led to a decrease in the efficiency of the purification systems. This can be verified by the results reported in the post-typhoon phase, where higher TTHM concentrations of drinking fountain water samples compared with analogous tap water samples were observed in some cases. It is possible that the filters in the drinking fountains became saturated as the target concentration from the intake water exceeded the system’s operational capacity, indicating desorption of some THMs from the purification structure. Poorly maintained drinking fountains, such as overused filters, can be a source of secondary contamination. This is particularly important in the wet season with a higher frequency of heavy rainfall and higher organic matter loading. To minimize this, drinking fountains require routine inspections that include keeping the filters working and replacing them regularly. Water filters that have been overused or gone unused for weeks should be replaced, and the system should be thoroughly cleaned. Moreover, as public drinking fountains might be outdated and overburdened, more precautious usage of drinking fountains in severe weather conditions with a high turbidity of intake water is highly recommended.

A higher percentage distribution of CHCl_3_ was found in drinking water compared with tap water samples, showing the efficiency of activated carbon filters commonly included in reverse osmosis systems in removing brominated THMs. Wang *et al.*^44^ showed that approximately 30–48% of THMs are brominated species in the finished water of different metropolitan areas of Taiwan, including Taipei, due to the presence of bromide in the raw water. On the other hand, NOM can influence the rate of bromide oxidation to bromate and consequently the formation of brominated THM^45^. Previous studies showed that the bromine (Br) level in paddy soils is generally lower than soils under forest or natural grassland, probably because the Br leached from paddy soils when they were flooded^46^. Changes in land use pattern, such as rice field cultivation and poor soil and water conservation measures along rivers, may lead to numerous mudslides during typhoon seasons, causing an increased bromide concentration in rivers. Therefore, a safe method for removing bromide in the drinking water in storm seasons is needed.

It has been shown that membrane filtration technologies can effectively and economically remove DBP precursors in NOM-laden waters, and reverse osmosis is a very good approach to eliminate brominated DBP^47^. The efficiency of granulated activated carbon is proportional to the number of bromine atoms in the molecule^48^. As chlorine atoms are replaced by bromine atoms, the molecular weight greatly increases, resulting in a higher removal efficiency^49^. The lower percentage distribution of CHClBr_2_ and CHBr_3_ in output water is then attributed to a higher molecular weight and brominating characteristics.

Boiling proved to be an important precautionary strategy to reduce THM formation in extreme weather conditions, as 70% or more of each THM species was removed upon boiling for only 5 min. Henry’s Law governs the THM removal process in boiling conditions (aeration with continuous agitation), controlling the equilibrium concentration of a compound in water in contact with air, or vice-versa. At 20 °C and 1 atmospheric pressure, chloroform has the highest Henry’s constant (0.1500) as it is the most volatile among the other THM species. As the bromine content of THMs increases, the Henry’s constant and correspondingly, the volatility of the compound decreases.

Although the mean concentration of THMs in water samples after the typhoon event was still below the regulated MCL for TTHM set by the Taiwan Environmental Protection Agency (80 μg/L), the results may provide an indication of the potential impacts on surface-based drinking water resources by future intense storm events. This is particularly important because a strong rising trend in a tropical cyclone with possibly the most intense (categories 4 and 5) systems has been shown in previous studies. As mentioned earlier, the EPA also intends to propose more limitations for THMs in drinking water, and there are even stricter regulations on levels in the drinking water of some other countries.

Brown *et al.*^50^ showed that hurricane Irene (2011) (category 3 hurricane) caused major increases in freshwater discharge and DOC levels compared with the pre-storm base-flow conditions in the Neuse River Estuary, NC, USA, and Yoon and Raymond^51^ reported an increased contribution of aromatic organic matter in New York after the same hurricane.

Nguyen *et al.*^21^ also showed a strong positive correlation between DBPFP and DOM optical measurements (UVA_254_ and fluorescence EEM peaks) in a forested watershed in Korea during storm events. They suggested a higher potential of humic-like DOM components on DBPs formation upon chlorination than other DOM moieties.

DOM levels rising over a short period of time with a temporary change in DOC quality can cause serious issues for water treatment. Higher organic matter input with lower processing operations suggest that allochthonous DOM will dominate over autochthonous types during storm events^52^. Therefore, immediate reciprocal action to DOM quality change, including monitoring and process optimization, is highly needed. In Taiwan and most of the Eastern Asian countries, typhoon rainfall accounts for a significant portion of the total annual rainfall. High loading of sediments and debris carried in stream flows during typhoon events may also cause heavy damage and serious water supply problems. Furthermore, the DOC trend seems to be increasing around the world^53^, and our results suggest that an increased frequency of typhoon events can be considered one of the driving forces responsible for the global DOC trend. Therefore, prediction and evaluation of the impact of climate changes on annual typhoon rainfall and storm intensity is critical for water resources management and a necessary addition to climate change impact studies.

## Methods

### Sample collection

**Figure 6:**
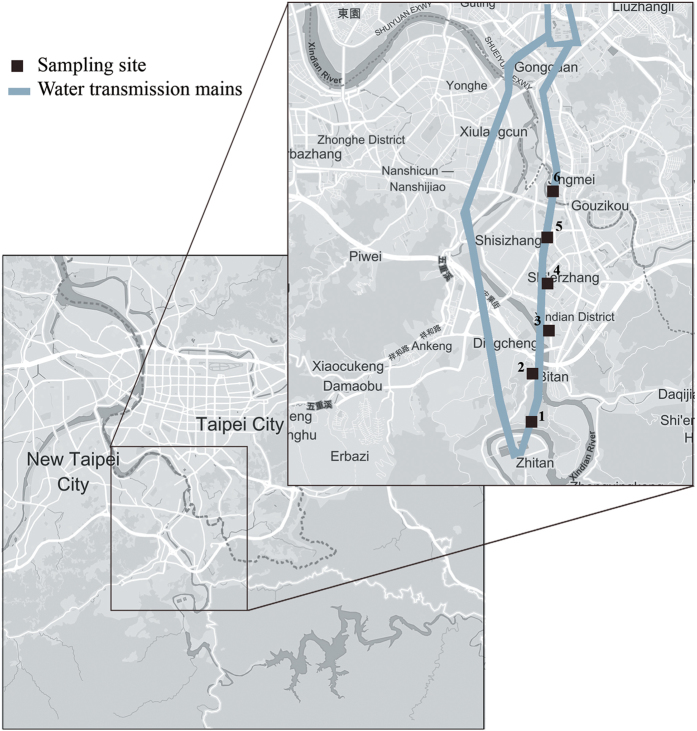
Map of the sampling locations (square marks). The map was generated using the Mapbox online platform (www.mapbox.com) (©Mapbox, ©OpenStreetMap).

The Taipei water treatment plant, which is the major tap water supplier in the Great Taipei Metropolitan Area, provides 2 × 10^6 ^m^3^ of drinking water per day and serves approximately 69% (3.8 million users) of the total residents in the area. The plant is governed by the water production department of the Taipei Water Company and consists of grit removal, prechlorination, coagulation, sedimentation, filtration, and post-chlorination as the purification processes^54^. The principal source of raw water collected by the Taipei Water Department (TWD) is the Xindian Creek, which represents 97% of total raw water supply. Two water intake units are in operation at the creek: the Qingtan Weir is located where water is conveyed by gravity to the Zhangxing and Gongguan Purification Plants for treatment, and the Zhitan Weir is located where its water is transmitted to the Zhitan Purification Plant^55^. Samples were collected from water transmission mains directly fed from the Zhitan purification plant, which presently supplies more than 70% of the water requirements of greater Taipei area. Tap and drinking water samples were collected two days before and after typhoon Soudelor hit Taipei City on August 8, 2015. The map of the sampling locations is shown in Fig. 6. The information about water transmission mains was obtained from TWD^55^. Samples were taken using a stratified random sampling technique following the Standard Methods for the Examination of Water and Wastewater^56^. The water distribution system in the typhoon-affected area was divided into 6 sites (strata) in which 8 random samples from each stratum were taken. The 48 total samples were then collected from tap or drinking fountain water before or after the typhoon event and the mean concentration of water quality parameters were reported for each sampling site. Additional samples were randomly taken to verify the sufficient number of samples in which the difference between additional and allocated samples in each strata were not significant (P-value ≥ 0.1).

Before collecting samples, the faucet was opened for approximately 5 min to ensure that the water was coming directly from the distribution system. For the THM analysis, water samples were collected in 250-mL glass bottles. The bottles were filled just to overflowing without passing air bubbles through the sample. They were previously washed with phosphate-free detergent, rinsed with tap water, deionized water and ultra-pure water, and placed in an oven at 400 °C for 1 h. Before sampling, a sodium thiosulfate solution (10%) was added to bottles (1.5 mL) to eliminate any remaining residual chlorine and to stop further THM formation. Additional 125 mL plastic bottles were used to collect samples for organic carbon and UV-absorbance measurements. Once collected, samples were stored in the dark at 4 °C and immediately transferred to the laboratory for analytical procedures. The boiling experiments were conducted on tap water samples in pre- and post-typhoon phases. Samples were boiled using a 2.5-quart tea kettle on a portable stovetop in the laboratory. The samples were allowed to cool down prior to extraction.

### Analytical Procedure

Chemicals including methanol, acetone, carbon disulfide, and 1,2-dibromopropane with purity higher than 99% were supplied by Merck chemical company (Merck, Darmstadt, Germany). As an indicator of humic acid content, UV-absorbance was measured on samples previously filtered through 0.45 μm membrane filters using UV/visible spectrophotometry (GENESYS™ 10S, WI, USA) at 254 nm with 50 mm optical path quartz cells. DOC was analysed using a TOC analyser (Aurora model 1030w /1088) after filtration through filter paper with a pore size of 0.45 μm. Standards of 0, 1, 2, 5, and 10 mg/L of total carbon were prepared with potassium hydrogen phthalate (KHP), using ultrapure water for all dilutions. pH and turbidity were measured on-site using a pH metre (Thermo Scientific Orion 4-star Benchtop pH metre), and a Hach turbidimeter (2100N model), respectively. The Centrifuge (Z32 HK, Germany) was used for centrifugation. SUVA at 254 nm, as an indicator of the humic content of water, was calculated by dividing a sample’s ultraviolet absorption at a wavelength of 254 nm (UV_254_) by its concentration of DOC.

A solution of the four THMs (TCM, BDCM, DBCM, and TBM) in methanol (2000 mg/L each) was purchased from Sigma-Aldrich (Milwaukee, WI, USA). Gas chromatographic separation and determination of the THMs was performed with a GC system (AGILENT 6850, USA) with a split/splitless injector and an electron capture detector. Compounds were separated on a 25 m × 0.32 mm capillary column coated with a 1.20 μm film of CP-Sil 13CB (86% methyl 14% phenyl siloxane). The oven temperature was maintained at 30 °C for 5 min then programmed at 10 °C min^−1^ to 140 °C which was held for 2 min. The ECD temperature was maintained at 300 °C. Ultrapure nitrogen (99.9999%, Air Products), used as a make-up gas for ECD, was passed through a molecular sieve trap and an oxygen trap (Crs, USA) at a flow of 30 mL/min.

### THM extraction procedure

**Figure 7:**
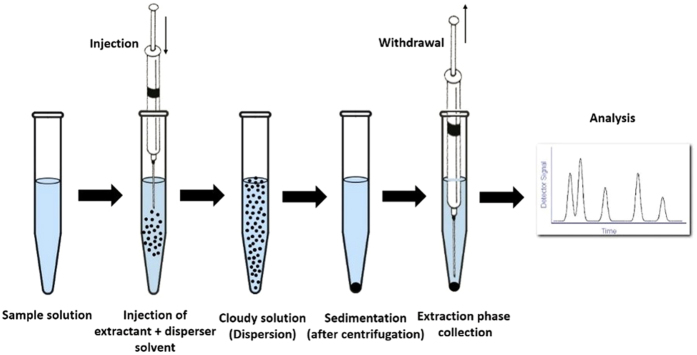
Schematic illustration (drawn by one of the co-authors) of DLLME.

The THM extraction process is based on dispersive liquid-liquid microextraction (DLLME), a simple and rapid pre-concentration and microextraction method that was developed by Kozani *et al.*^57^ with a slight modification. It is an environmentally friendly sample preparation method with highly promising target analytical potential (Fig. 7).

A 5.00 mL water sample was placed in a 10-mL screw cap glass test tube with a conical bottom and spiked with 1,2-dibromopropane as the internal standard. Acetone (0.50 mL) as disperser solvent containing 20.0 μL carbon disulfide (extraction solvent) was rapidly injected into the sample solution using a 0.50-mL syringe (gas-tight; Hamilton, USA). A cloudy water–acetone–carbon disulfide mixture was formed in the test tube. The mixture was gently shaken and then centrifuged for 1.0 min at 6,000 rpm. The dispersed fine particles of the extraction phase settled to the bottom of the conical test tube, of which approximately 1 μL was injected into the GC.

### Statistical analysis

Statistical analysis, including Pearson and Spearman’s rank correlations, was carried out using SPSS (version 16.0, SPSS Inc., Chicago, IL, USA) software to assess the relationships between water quality parameters and THM concentration. The Pearson’s product momentum correlation coefficient (*r*_*p*_) was used to estimate linear correlations. The value of *r*_*p*_ was always between −1 (perfect negative correlation) and +1 (perfect positive correlation), and 0 indicated the absence of a relationship. Spearman’s rank correlation is a nonparametric alternative to the correlation that does not rely on assumptions of normality and is less sensitive to outliers than standard Pearson correlation. In conducting a correlation analysis, Spearman’s rank coefficient can be calculated to compare with Pearson’s correlation. If the value is fairly similar to the Pearson’s correlation coefficient, it indicates that the outliers are not substantially influencing the results^58^. Correlations are considered statistically significant at a 95% confidence interval (P < 0.05).

## Additional Information

**How to cite this article**: Fakour, H. *et al.* Impacts of Typhoon Soudelor (2015) on the water quality of Taipei, Taiwan. *Sci. Rep.* **6**, 25228; doi: 10.1038/srep25228 (2016).

## Figures and Tables

**Table 1:** TTHM concentrations (μg/L) in tap and drinking water fountain samples during pre- and post- Typhoon Soudelor (2015).

Chemical	Water type	Phase	Site No.					
			1	2	3	4	5	6
TTHMs	Tap	Pre-	15.9	12.1	18.5	17.5	24	32.8
		Post-	23.1	25.3	37.2	36.5	40.8	64.9
	Drinking	Pre-	17.3	11.6	15.1	18.2	14.5	25.8
		Post-	19.1	18.7	27.5	42.1	43.2	56.1

**Table 2:** Spearman’s rank (above the diagonal) and Pearson’s (below the diagonal) correlation matrix for water quality parameters and THMs concentration.

	pH	DOC	SUVA	Turbidity	THM
pH	1	−0.038	−0.07	−0.01	−0.19
	(1)**	(−0.72)*	(−0.81)*	(−0.79)*	(−0.83)*
DOC	−0.04	1	0.69*	0.67*	0.51*
	(−0.75)*	(1)	(0.95)*	(0.89)*	(0.72)*
SUVA	−0.05	0.72*	1	0.11	0.55*
	(−0.79)*	(0.96)*	(1)	(0.84)*	(0.81)*
Turbidity	−0.002	0.61*	0.19	1	0.12
	(−0.82)*	(0.92)*	(0.90)*	(1)	(0.88)*
THMs	−0.21	0.53*	0.52*	0.18	1
	(−0.81)*	(0.76)*	(0.80)*	(0.93)*	(1)

^*^Significant at P-value < 0.05.

^**^Values in parentheses representing post-typhoon sampling stage.
